# Smoking Cessation Interventions and Abstinence Outcomes for People Living in Rural, Regional, and Remote Areas of Three High-Income Countries: A Systematic Review

**DOI:** 10.1093/ntr/ntad098

**Published:** 2023-06-20

**Authors:** Joshua Trigg, Eliza Skelton, Alistair Lum, Ashleigh Guillaumier, Kristen McCarter, Tonelle Handley, Lucy Judd, Alexie Lye, Billie Bonevski

**Affiliations:** Flinders Health and Medical Research Institute, College of Medicine and Public Health, Flinders University, Adelaide, SA, Australia; Faculty of Health and Medicine, School of Medicine and Public Health, University of Newcastle, Callaghan, NSW, Australia; Faculty of Health and Medicine, School of Medicine and Public Health, University of Newcastle, Callaghan, NSW, Australia; Flinders Health and Medical Research Institute, College of Medicine and Public Health, Flinders University, Adelaide, SA, Australia; Faculty of Health and Medicine, School of Medicine and Public Health, University of Newcastle, Callaghan, NSW, Australia; Faculty of Health and Medicine, School of Medicine and Public Health, University of Newcastle, Callaghan, NSW, Australia; Faculty of Health and Medicine, School of Medicine and Public Health, University of Newcastle, Callaghan, NSW, Australia; Faculty of Health and Medicine, School of Medicine and Public Health, University of Newcastle, Callaghan, NSW, Australia; Flinders Health and Medical Research Institute, College of Medicine and Public Health, Flinders University, Adelaide, SA, Australia

## Abstract

**Introduction:**

Tobacco smoking rates in high-income countries are greater in rural, regional, and remote (RRR) areas compared to cities. Yet, there is limited knowledge about interventions targeted to RRR smokers. This review describes the effectiveness of smoking cessation interventions for RRR smokers in supporting smoking abstinence.

**Aims and Methods:**

Seven academic databases were searched (inception-June 2022) for smoking cessation intervention studies to include if they reported on RRR residents of Australia, Canada, or the United States, and short- (<6 months) or long-term (≥6 months) smoking abstinence outcomes. Two researchers assessed study quality, and narratively summarized findings.

**Results:**

Included studies (*n* = 26) were primarily randomized control (12) or pre-post (7) designs, from the United States (16) or Australia (8). Five systems change interventions were included. Interventions included cessation education or brief advice, and few included nicotine monotherapies, cessation counseling, motivational interviewing, or cognitive behavioral therapy. Interventions had limited short-term effects on RRR smoking abstinence, decreasing markedly beyond 6 months. Short-term abstinence was best supported by contingency, incentive, and online cessation interventions, and long-term abstinence by pharmacotherapy.

**Conclusions:**

Cessation interventions for RRR smokers should include pharmacotherapy and psychological cessation counseling to establish short-term abstinence, and identify effective means of maintaining abstinence beyond 6 months. Contingency designs are a suitable vehicle for psychological and pharmacotherapy support for RRR people who smoke, and intervention tailoring should be explicitly considered.

**Implications:**

Smoking disproportionately harms RRR residents, who can encounter access barriers to smoking cessation support. High-quality intervention evidence and outcome standardization are still required to support long-term RRR smoking abstinence.

## Introduction

Smoking cessation is central to preventing chronic health conditions through reducing stroke or acute myocardial infarction risk,^1^ improving cancer-related treatment and ­recovery outcomes,^2,3^ and reducing overall mortality rates. High-income countries like Australia, Canada, and the United States have well-resourced tobacco control measures, aligned with World Health Organization (WHO) Framework Convention on Tobacco Control strategy.^4^

However, smoking rate reductions vary between and within countries. Rural, regional, and remote (RRR) areas can have higher smoking rates compared to metropolitan areas.^5,6^ In the United States, combined data (2007–2014) showed higher current cigarette use in rural (27.3%) compared to the urban (21.3%) areas.^5^ Australia, had an increasing daily age-standardized daily smoking prevalence rates between major cities (12.8%), inner regional (16.5%), and remote areas (19.6%) in 2017–2018.^7^ Elsewhere, international prevalence estimates also indicate disparities (eg, rural Appalachia, 33%^8^; Canadian Northwest-Territory, 25%^9^). Despite higher smoking rates in RRR areas, research shows that these smokers are generally motivated to quit, with a high proportion having already made several quit attempts.^6,10^

Best practice in smoking cessation support involves providing both behavioral counseling and pharmacotherapy.^11^ Yet, surveys of rural smokers show a significantly lower likelihood of smoking cessation advice provision compared to urban counterparts.^12^ Rural residents more often face geographical and socioeconomic barriers to accessing smoking cessation support.^13,14^ As rural-living Indigenous peoples have higher all-cause mortality,^15^ and smoking rates than non-Indigenous peoples,^16^ this further underscores the need for increased RRR-specific interventions^17^

Limited evidence is available for the types of smoking cessation interventions preferred and the delivery format (eg, in-person, telehealth) for smokers residing in RRR areas and their effectiveness.^18^ Preliminary studies indicate that telehealth and web-based intervention modalities can reduce access barriers to evidence-based smoking cessation support.^18^ There is a clear need to synthesize current literature specific to RRR smokers to identify effective smoking cessation interventions. This review focuses on Australia, Canada, and the United States, given their comparable economic systems, geographical size, and smoking rates. This review aimed to describe the types of smoking cessation interventions for RRR smokers, describe short (<6 months) and long-term (>6 months) smoking cessation outcomes. This review followed Preferred Reporting Items for Systematic Reviews and Meta-Analyses guidelines,^19^ and a protocol (PROSPERO: CRD42020177398).^20^

## Methods

### Search

Medline, EMBASE, PsycINFO, CINAHL, Cochrane, Informit Health, and Scopus were searched for literature published from database inception to June 2022, and reference lists of relevant studies were searched. Search terms addressed tobacco use, cessation, remoteness, and intervention design (Supplementary Material 1).

### Inclusion Criteria

Studies needed to be conducted in RRR areas of Australia, Canada, and the USA, as their comparable high-income status, geographic size, smoking rates, RRR population proportions, and tobacco control objectives help generalizability of findings^20^ Studies also needed to include adult tobacco smokers, a smoking cessation intervention (behavioral, pharmacological, or complementary), an outcome measure of smoking cessation such as self-reported abstinence or biochemically verified abstinence (eg, continuous abstinence [CA], point-prevalence abstinence [PPA]) either <6 months or ≥6 months following the intervention, or smoking-related behavior (eg, reduced cigarette smoking or cigarettes smoked per day), and had to employ randomized controlled trial (RCT), cluster-RCT (C-RCT), pre-and post-test, or related intervention designs (eg, prospective follow-up). We included studies that self-classified as being set in RRR areas, commonly based on road distance^21^ or travel time^22^ to the nearest urban center, or population density.^23^

### Study Quality

Methodological quality was assessed by two researchers using the Scottish intercollegiate guidelines network risk assessment, and overall quality of evidence using the grades of recommendation, assessment, development, and evaluation (GRADE).

### Analysis

**Figure 1.**
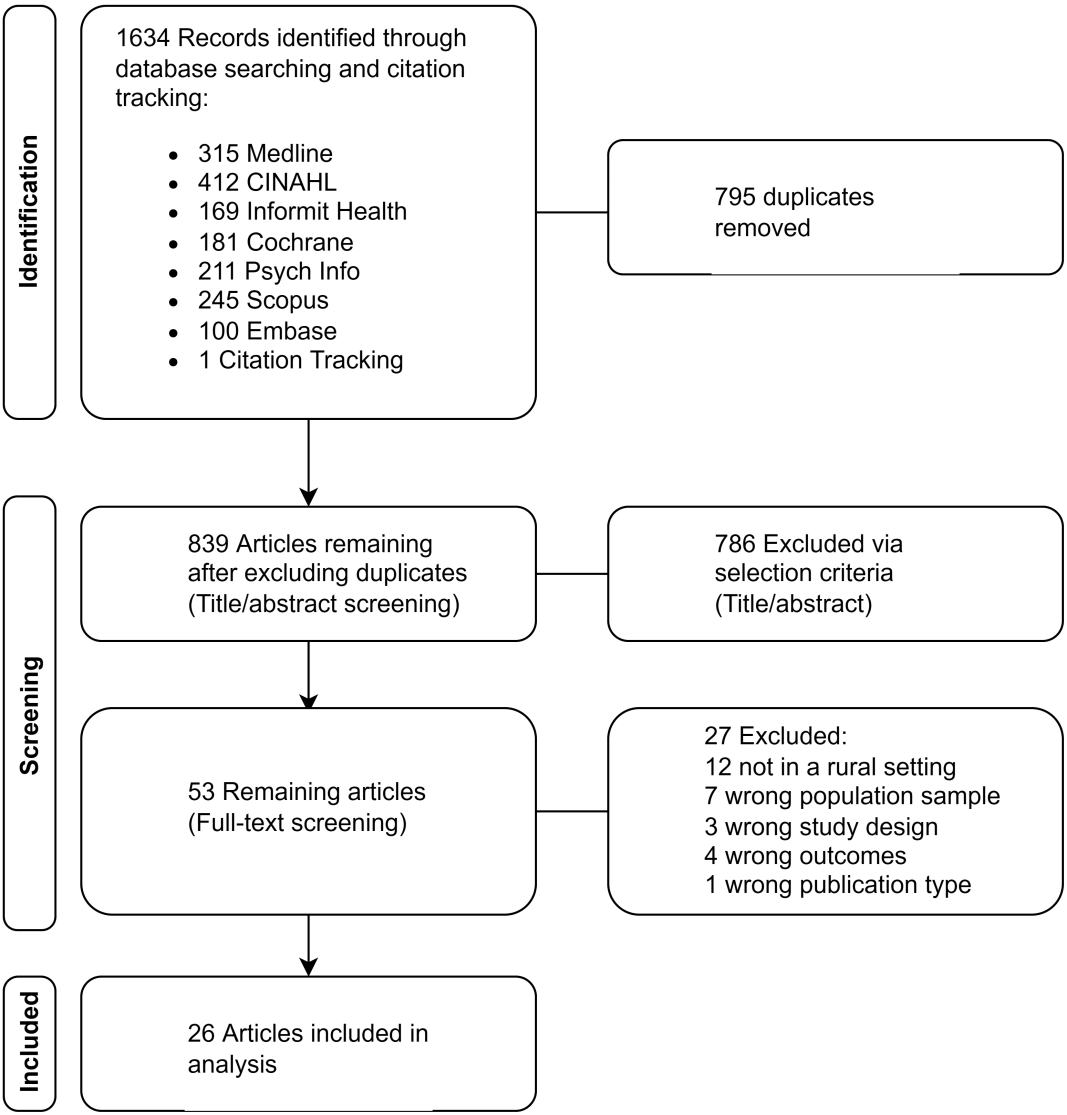
Preferred Reporting Items for Systematic Reviews and Meta-Analyses search stages for article identification and inclusion.

After title and abstract screening, full papers were screened for relevance to review aims (AL, LJ, and TH). Exclusion reasons (Figure 1), and discrepancies were discussed. Data extraction used a custom tool, piloted on 5% of full texts for reviewer consistency by two researchers (90% agreement), to record study details: Author, year, country, population, design, program name, sample characteristics, retention rate, intervention, and comparator/control-group descriptions, abstinence and smoking-related outcome measures, intervention adherence, study, and intervention duration. Findings were narratively synthesized by three authors (AL, JT, BB), and descriptive statistics were used to report study characteristics. Outcome measures (eg, 7-day PPA, 30-day PPA, and CA) and follow-up time points were grouped to examine short-term cessation (<6 months) versus long-term abstinence (≥6 months) for intervention types. For brevity, we hereafter refer to people who smoke as “smokers” noting that this is not a sole defining characteristic.

## Results

### Study Characteristics

Titles were screened following database searching (*n* = 839), with 786 excluded via title and abstract review, and 53 articles full text reviewed, providing 26 final articles for extraction (Figure 1). Additional details were requested for six studies.^18,24–28^ Study population, design, duration, and behavioral and pharmacological intervention characteristics are summarized in Table 1 and detailed further in Supplementary Material 2. Most studies were conducted in the United States (*n* = 16) followed by Australia (*n* = 8) and Canada (*n* = 2). Most study designs were RCT (*n* = 11), C-RCT (*n* = 3), and pre- and post-test (*n* = 7), with three prospective follow-ups, one between-subjects mixed-method program evaluation, and one quasi-experimental control-group design. Some designs were also systems change interventions.^24,25,29–31^

**Table 1.** Summary of Tobacco Smoking Cessation Intervention Characteristics With Selected Short- and Long-Term Smoking Abstinence Treatment Outcomes (*N* = 26)

Study	Design/population	Intervention	Smoking abstinence outcomes^*^	
			<6 months	≥6 months
Adams 2006–AUS	Pre-post, Aboriginal Australian, *n* = 32	Aboriginal health service program with pharmacotherapy access.	3 weeks: 19.0%	—
Azor Hui 2013–United States	Follow-up, rural smokers, *n* = 333	3-arm: Telephone counseling and pharmacotherapy access.	—	36 months: ≤4.3%
Bailey 2015–United States	RCT, rural pregnant women, *n* = 1947	Psychoeducation and brief quit intervention.	—	26 weeks: 28.1%
Bottorff 2016–CAN	Between-subjects, rural, *n* = 240	Education in the 5As, online and healthcare provider resources.	—	1 year: 7.8%
Breen 2021–AUS	Follow-up, rural smokers, *n* = 62	Pharmacy-delivered brief advice and phone support with incentives.	3 months: 19.4%	—
Britton 2006–United States	Quasi-exp, pregnant women, *n* = 194	Tailored health messaging on quitting, and cessation guidance.	16 weeks: 26.0%	28 weeks: 25.0%
Bullock 2009–United States	RCT, pregnant women, *n* = 695	4-arms, with nurses trained in support, and resource provision.	—	6 weeks postbirth,11.4%–13.5%
Byaruhanga 2021–AUS	RCT, rural smokers, n=655	3-arm: Video and phone counseling vs. written resources.	4 months: 4.4-18.9%	—
Carlson 2012–CAN	Follow-up, rural smokers, *n* = 554	Cessation counseling with clinical psychologists.	3 months: 25.5%	Month 6 (14.1%), 12 (25.5%)
Ellerbeck 2009–United States	RCT rural smokers, *n* = 750	Cox et al. (2008) design, used in Azor Hui et al. (2013)	—	24 months: 13.5%–14.8%
Ferketich 2014–United States	C-RCT, Appalachians, *n* = 214	Physician education (5As), resources, and clinic support staff.	3 months: 11.0-24.2%	—
Gould 2015–AUS	Pre-post, rural smokers, *n* = 42	Single-session behavioral change education and NRT samples.	—	6 months: 14.3–28.6
Hancock 2001–AUS	RCT, rural Australians, *n* = 10 256	Systems intervention for workplace smoke-free environments.	—	Post intervention = 20.4%
Harris 2015–United States	RCT, Pregnant Appalachians, *n* = 17	2-arm: Online contingency program and phone counseling support.	3 months: 20.0%–28.6%	7–8 months: 14.3%–30%
Horn 2004–United States	C-RCT, rural teens, *n* = 258	Gender-specific cessation support groups vs. brief advice.	12 weeks: 1.7%–8.3%	—
Ivers 2003–AUS	Pre-post, Aboriginal Australian, *n* = 111	Brief advice with counseling, resources, and nicotine patches.	—	6 months: 3.0%–10.0%
Marley 2014–AUS	RCT, Aboriginal Australians, *n* = 168	In person motivational quit counseling vs. brief advice.	—	Month 6 (9.0%), 12 (11.0%–29.0%)
Northridge 2008–United States	Pre-post, rural Appalachians, *n* = 725	Behavior change program, group support, and pharmacotherapy.	2 months: 51.4%	12 months: 17.0%
Reynolds 2015–United States	RCT, rural Appalachian youth, *n* = 62	Abstinence contingency management vs. non-contingency	32 days: 0.0%	—
Richter 2015–United States	RCT, Kansan patients, *n* = 566	2-arm: Telemedicine or phone counseling with pharmacotherapy.	—	12 months: 9.8%–12.0%
Santiago-Torres 2022–United States	RCT, rural U.S. smokers, *n* = 550	2-arm: iCanQuit (accept/commit) vs. QuitGuide (advice) apps.	3 months: 15%–26%	Month 6 (35.0%) 12 (15.0%–35%)
Schorling 1997–United States	C-RCT, African Americans, *n* = 896	Church-designed cessation counseling vs. hypertension advice.	—	18 months: 9.6%
Sheffer 2004–United States	Pre-post, Arkansan patients, *n* = 1644	Specialist-led individual and group tobacco-treatment sessions.	3 months: 42.0%	—
Sheffer 2009–United States	Pre-post, rural Arkansans, *n* = 2350	Specialist CBT and relapse prevention sessions with optional NRT.	3 months: 19.0%–28.6%	12 months: 13.0%–24.3%
Stoops 2009–United States	RCT, Appalachian residents, *n* = 68	2-arm: Online contingency program vs. non-contingent control.	1–6 weeks 30.4%–33.9%	—
Wong 2004–AUS	Pre-post; rural patients, *n* = 18	Prescription bupropion SR was provided for 8 weeks.	Month 2 (38.9%) 3 (38.9%)	—

^*^Further outcomes indicators available in Supplementary Materials, with the highest treatment condition outcome timeframe shown for each category. An outcome range is reported where multiple treatment conditions were specified. Further outcome figures are available in Supplementary Materials. NR = not reported; ITT = intention to treat; CC = complete case; CA = complete abstinence; verified = biochemically verified. Reynolds (2015) refers to cigarettes per day and biochemical verification values. C-RCT = cluster-RCT; NRT = nicotine replacement therapy.

Intervention duration ranged from <1 day^32^ to 24 months^26^ when reported (n=25). Samples ranged from 17 to 10 256 participants, and retention from 11% to 100%. Average participant age ranged from 16.2 years^33^ to 55.0 years old,^24^ with samples comprising more females (eg, 59.3%^24,30,34^ to 100%^25,29,35^), more having completed high school,^18,24,26,27,30,34,36,37^ in employment^24,27,36–38^ and of Caucasian ethnicity (eg, 82.6%^27^ to 95%^29^).

### Quality Appraisal

No study received a Scottish intercollegiate guidelines network high-quality methodological rating, nine studies received an acceptable rating,^18,25,26,28,29,38–41^ 17 studies received a low-quality rating,^24,27,30–37,42–48^ and no studies were rated unacceptable (Table 2). A common reason for a low-quality rating was low detail on measurement and minimization of potential confounders. Overall GRADE rating of the certainty of the body of evidence reported in this review was low, and as assessment methods for key outcomes (eg, cessation) were heterogeneous, this introduced high bias risk.

**Table 2.** Ratings of Methodological Quality of Included Studies: Unacceptable/Reject, Low Quality, Acceptable, High Quality as Per the Scottish Intercollegiate Guidelines Network Risk Assessment

Study	Overall quality assessment
Adams et al.^42^	Low quality
Azor Hui et al.^43^	Low quality
Bailey^35^	Low quality
Bottorff et al.^24^	Low quality
Breen^34^	Low quality
Britton et al.^29^	Acceptable
Bullock et al.^25^	Acceptable
Byaruhanga et al.^40^	Acceptable
Carlson et al.^18^	Acceptable
Ellerbeck et al.^26^	Acceptable
Ferketich et al.^30^	Low quality
Gould and Watters^32^	Low quality
Hancock et al.^31^	Low quality
Harris and Reynolds^44^	Low quality
Horn et al.^33^	Low quality
Ivers et al.^45^	Low quality
Marley et al.^38^	Acceptable
Northridge et al.^36^	Low quality
Reynolds et al.^39^	Acceptable
Richter et al.^27^	Low quality
Santiago-Torres et al.^41^	Acceptable
Schorling et al.^37^	Low quality
Sheffer and O’Bannon^46^	Low quality
Sheffer et al.^47^	Low quality
Stoops et al.^28^	Acceptable
Wong and Fraser^48^	Low quality

### Study Findings

The following sections present intervention characteristics and outcomes according to short-term (<6 months) and long-term (≥6 months) smoking abstinence, for different intervention types: Contingency/incentives and online; training health providers; psychological/pharmacological combination; telehealth; education programs; nicotine replacement therapy (NRT)/stop-smoking medications. The most frequent intervention types provided education, resources, and feedback to promote smoking cessation care, including smoking cessation care, and designating a cessation support person. As systems change interventions in this review (*n* = 5) used multiple components for which individual effects are difficult to interpret, these are discussed in a separate section. Design and abstinence outcomes are presented in greater detail in Supplementary Materials 2 and 3 and are discussed in the following sections.

### Contingency/Incentives and Online

#### Short-Term Abstinence

In a 6-week home-based program (Reynolds et al.^39^), Appalachian adolescents received financial vouchers for three webcam-verified daily carbon monoxide (CO) samples indicating smoking abstinence. CO reductions were financially reinforced in treatment, and timelines were reinforced in the control condition. Treatment reduced CO from baseline to end-of-treatment by more (4.4 ppm, *p* < .0001) than did control (1.7 ppm, *p* = .06). Average daily cigarettes also decreased in both conditions, though no between-group abstinence effects were found.

Online abstinence-contingent management was further supported with Appalachian adults in a 6-week program reinforcing twice-daily CO samples, compared to non-reinforced controls (Stoops et al.^28^). An online display showed daily results, cumulative CO, and money earned. Smokers in the contingency condition were significantly more likely than controls to achieve CA from weeks 1 (37.1% vs. 6.1%), to 6 (2.9% vs. 0.0%), with no controls abstinent after week 1. Short-term abstinence was achieved but not maintained, and NRT was not restricted.

A separate program with pregnant Appalachian smokers compared 6-week online financial-contingency management to five cessation phone counseling calls (Harris et al.^44^). Short-term CA from 3.5 to 5.5 months was supported via both contingency (14.3% to 28.6%) and phone (10.0% to 20.0%), with online delivery considered feasible. This aligns with a pharmacy-delivered program providing seven brief advice sessions to rural Australian female smokers, with abstinence-contingent payments over 3 months (Breen et al.^34^). This decreased average daily cigarettes at week 1 (*M* = 17.46, to *M* = 2.60, *p* < .001), though CO-verified 7-day PPA decreased from week 1 to 3-month post-enrollment (32.25% to 19.35%, untested).^34^

#### Long-Term Abstinence

Although most contingency-based interventions were short-term abstinence-focused, Harris et al,^44^ assessed abstinence at three long-term points: Contingency (6.86, 8.05, and 8.75 months), phone (6.45, 7.37, and 8.19 months). Cotinine-verified 7-day PPA decreased in the online contingency (28.6% to 14.3%, untested) but not in phone counseling condition (30.0% to 30.0%) from weeks 4 to 6 post-enrollment.

### Training Health Providers

#### Short-Term Abstinence

A study training regional tobacco-treatment specialists to provide preoperative smoking cessation support to Arkansan smokers found a large self-reported quit rate for patients completing the program, versus all patients (74.0% vs. 42.0%) (Sheffer et al.^46^). Yet, this rate decreased for both participants and all patients 3 months after discharge (42.0% vs. 30.0%). A separate study at 20 healthcare sites delivered six sessions of relapse-focused cognitive behavior therapy (CBT) to rural low-SES Arkansan smokers (Sheffer et al.^47^), returning a low 7-day PPA 3 months after intervention (ITT, 18.95%).

In a pre-post design study of an 8-week multicomponent smoking cessation intervention for rural smokers, (Northridge et al.^36^), health clinic staff and volunteers were trained to integrate cessation support from the American Lung Association into clinical consultations. Program completers were significantly more likely than non-completers to have quit smoking following intervention (AOR = 7.98, 95% CI = 4.87, 13.08).

#### Long-Term Abstinence

In Schorling et al.,^37^ church volunteers trained in stages-of-change counseling and self-help smoking cessation material were compared to volunteers trained in hypertension counseling (control) for supporting rural African American smokers to quit in 70 clinics. Self-reported 30-day PPA was low for treatment (6.20%) and control (9.60%) after 18 months intervention.

Long-term cessation was supported by smokers aged ≥16 years at two north–western Australia Aboriginal health ­services (Marley et al.^38^). Usual care (ie, routine advice, pharmacotherapy) was compared to usual care plus 12 Aboriginal-led counseling sessions over 12 months. Self-reported 7-day PPA at 6 months post-enrollment did not differ between conditions (11.0% vs 9.0%). At 12 months post-enrollment, cotinine-verified 7-day PPA was higher in the intervention group compared to controls (29.0% vs. 8.0%), though not for ITT.

In Northridge et al.,^36^ having health providers deliver cessation support to Appalachian smokers led to a self-reported quit rate of just 17% after 12 months. Low long-term abstinence was also seen in Sheffer and Bannon.^47^ Here, training specialists to deliver tobacco dependence and relapse prevention CBT to Arkansan smokers produced comparably low 7-day PPA after 12 months (24.27%) and lower for ITT.

### Psychological/Pharmacological Combination

#### Short-Term Abstinence

Smoking abstinence was supported for Koori Australian smokers in a program combining Aboriginal health worker-led 3-week behavioral group education, Quitline referral and call, and doctor consultation, with NRT and buproprion ­information.^42^ Three-week PPA was high for this priority population despite absent co-design (19%), and no pharmacotherapy dosage description.

In the Northridge et al,^36^ programs for health providers delivered cessation support to Appalachian smokers, both behavior modification and 8 weeks of free NRT or buproprion were provided with follow-up group support. However, self-reported quitting was higher in urban compared to rural (60.6% vs. 51.4%, untested) smokers, with rural smokers less likely than urban to have quit. This supports Sheffer et al.,^47^ where six CBT quit sessions combined with 4 weeks of free NRT patches (dosage unspecified) for low socioeconomic status (SES) Arkansan smokers led to a moderate 7-day PPA 3-month post-intervention (28.6%). Approximately 51.1% of participants used patches, with 15.9% using other pharmacotherapy (eg, bupropion).

#### Long-Term Abstinence

A study comparing pharmacological management to moderate and high disease management conditions via motivational interviewing found no difference in 7-day PPA at 24 months between conditions when self-reported (23.0% to 27.9%), and cotinine verified (13.5% to 14.8%) (Ellerbeck et al.^26^). Although the difference was also not apparent at 36 months, varenicline use predicted higher likelihood of 7-day PPA.^43^

In Gould and Watters’,^32^ single-session CBT behavior-change training was provided to rural smokers in primary care, covering cessation reasons, planning, pharmacology, and pharmacotherapy. Follow-up 6 months post-intervention found low self-reported (28.6%) and biochemically verified (14.3%) quitting.

A further study compared integrated video telemedicine to telephone counseling for smoking cessation by low-SES rural Kansan smokers at primary care clinics.^27^ Similarly, counselors combined motivational interviewing and CBT, with telemedicine participants using clinic computers, and telephone participants on their own devices. Both conditions provided pharmacological advice. More smokers in the telemedicine condition used cessation medication (55.9% vs. 46.1%), though dosage was not described. Cotinine verified that 7-day ITT PPA at 12 months did not differ between telemedicine and telephone (9.8% vs. 12.0%).

In a 6-week program via Aboriginal health services,^45^ Aboriginal Australian smokers in the Northern Territory, received either pharmacological aid (decreasing dosage NRT patches plus brief advice) or brief advice only (ie, quit planning). At 6 months post-baseline, CO-verified PPA was low in both conditions (3.0% vs. 10.0%, untested), with NRT side effects in 29% of patch users. The Northridge et al.^36^ intervention also paired educational and pharmacological intervention for health provider-delivered cessation support. However, short-term abstinence effects were not sustained at 12 months (53.1% to 17.0%). Long-term abstinence was, however, supported by Sheffer and Bannon,^47^ where six sessions of smoking cessation CBT with a 4-week supply of NRT patches sustained abstinence at 12 months (CC, 24.27%), with self-efficacy a predictor of this.

### Telehealth

#### Short-Term Abstinence

A behavioral intervention by Breen et al.^34^ incorporated telephone cessation support access. However, among those obtaining further support (74.2%), the proportion accessing telephone support versus other supports (eg, varenicline, 19.6%; single NRT, 37.0%) was not specified. In a separate study of rural and urban Canadian smokers comparing in-person health service support to delivery via telehealth,^18^ eight group sessions were run over 4 months by psychologists addressing cessation education, self-monitoring, and relapse prevention. Three months into the intervention, ITT CA did not differ between on-site (27.3%) and telehealth (25.5%).

Short-term telehealth intervention feasibility was supported with pregnant Appalachian smokers,^44^ when comparing web-based contingency management for smoking cessation to a nurse-delivered phone counseling program. Feasibility of self-reported smoking abstinence from 3.5 to 5.5 months was supported for both contingency (14.3%–28.6%) and phone (10.0%–20.0%) conditions, given the small sample and untested differences.

Telehealth video counseling also supported short-term smoking abstinence for rural Australian smokers, via a program comparing 1 week of brief CBT and motivational interviewing with a trained quit advisor against telephone counseling.^40^ From baseline to 4 months, 7-day PPA was higher for video (18.9%) than phone (12.7%) counseling. Short-term abstinence was also supported by rural U.S. smokers using a mobile application presenting eight sets of acceptance and commitment therapy exercises.^41^ When compared to a smoke-free guidance app for increasing quit motivation, the acceptance and commitment therapy app produced higher 7-day PPA (26.0% vs. 17.0%) and 30-day PPA (15.0% vs. 9.0%) at 3 months.

#### Long-Term Abstinence

The acceptance and commitment therapy exercises mobile application tested with rural U.S. smokers above also suggested higher long-term abstinence outcomes over the smoke-free guidance app for 7-day PPA at 12 months (35.0% vs. 31.0%), and 30-day PPA at 12 months (29.0% vs. 25.0%), and CA at 12 months (15.0% vs. 10.0%).^41^ Azor Hui et al.^43^ showed also that rural Kansan smokers receiving pharmacotherapy (NRT patch or buproprion), low disease management, or high disease management pairing pharmacotherapy with motivational interviewing had comparably low 7-day PPA 36 months after intervention (5.3% vs. 3.7% vs. 4.3%). The Ellerbeck et al.^26^ studies comparing pharmacological to moderate and high disease management did not support long-term abstinence, as 7-day PPA at 24 months, was low in all three conditions, and lower again when cotinine-verified. Following the 24-month intervention, smokers more often reported abstinence in the combined disease management conditions compared to pharmacotherapy.

Lack of support for long-term abstinence was shown in Richter et al,^27^ when comparing video consultation to phone counseling. Biochemically verified 7-day ITT PPA at 12 months in rural Kansan smokers did not differ between video consultation (9.8%) and phone counseling (12.0%).

### Education Programs

#### Short-Term Abstinence

The Adams et al.^42^ smoking cessation program delivered with Koori Aboriginal Australian smokers included a short course education component with Quitline support, and produced a 19% 3-week PPA post-intervention.

In the Carlson et al^18^ studies, telehealth educational intervention produced comparable 3-month CA to in-person at 3 months (25.5% vs. 27.3%), suggesting high suitability for rural smokers. Both approaches used education in self-monitoring, smoking behaviors, cessation aids, withdrawal, stress, and relapse prevention.

Educational intervention also worked well with rural American teenagers,^33^ comparing multicomponent gender-specific sessions on smoking reasons, quit preparation, addiction, and social supports, to brief generic sessions giving quit advice. From baseline to 3 months post-intervention, CO-verified quitting was higher in the intervention than in control (8.33% vs. 1.67%). Verified quitting differences were not seen for females but not males, with gender interaction lowering effectiveness for males. Finally, the Northridge et al.^36^ studies of rural American smokers described earlier used an educational component from an American Lung Association program. Compared to smokers finishing in the first half of the program, those finishing in the second half were more likely to report quitting post-intervention.

#### Long-Term Abstinence

Bailey et al.^35^ compared four brief psychoeducation sessions to usual care in quitting for Appalachian pregnant smokers at prenatal practices. Sessions considered environmental, social, and motivational factors for abstinence. Intervention lasted from first antenatal visit to delivery, and abstinence at the end of the second trimester was significantly higher when using psychoeducation (28.1% vs. 9.8%).

Educational components were also present in Carlson et al.^18^ within eight group education sessions covering self-monitoring, smoking habits, withdrawal, stress management, and relapse prevention, comparing in-person against telehealth intervention for rural Canadian smokers. As noted earlier, abstinence did not differ between conditions at 6 or 12 months into the intervention, and effect of education could not be isolated.

In a brief behavior-change intervention, Gould and Watters^32^ used CBT education on quitting, ­pharmacological mechanisms, and cessation planning to support low self-reported (28.6%) and verified (14.3%) abstinence at 6 months in rural Australian smokers. Community education also received low support in Schorling et al.,^37^ where training volunteers in quit readiness counseling, compared to hypertension counseling, across 70 church-partnered clinics found no difference in self-reported 30-day PPA at 18-month follow-up (6.2% vs. 9.6%) among rural African American smokers.

### NRT and Stop-Smoking Medications

#### Short-Term Abstinence

A small study of rural Australian general, surgical, and drug treatment patients suggested feasibility of 8-week buproprion treatment for smoking cessation.^48^ Two months post-treatment commencement, self-reported PPA was moderately high (38.9%). Feasibility for short-term abstinence was also supported among Aboriginal Australian and Torres Strait Islander smokers, when using single-session brief advice control (health, quit readiness/planning) with 10 weeks of NRT.^45^ Participants had higher 6-month verified PPA than did controls (15.0% vs. 1.0%, untested), and more often reported reduction tobacco use (76.0% vs. 51.0%). However, with low NRT compliance, feasibility only is supported. Gould and Watters’^32^ study also provided smokers samples of patches, gum, or lozenges, though NRT contribution to abstinence was unreported.

#### Long-Term Abstinence

In Ellerbeck et al.,^26^ as described earlier, pharmacotherapy alone (6-week NRT 21mg patch or 7-week buproprion 2 × 150 mg) produced a lower 7-day PPA at 24 months when cotinine-verified, as combining pharmacotherapy with motivational interviewing produced more long-term abstinence. Under the same parent study^26^ Azor Hui et al^43^ found that recalcitrant (≥10 cigarettes daily) rural Kansan smokers had similarly low 7-day PPA 12 months after the 24-month intervention whether using pharmacotherapy (5.3%) or pharmacotherapy with counseling (3.7%–4.3%).

### Systems Change Interventions

#### Short-Term Abstinence

In a systems change program for Appalachian smokers (Ferketich et al.^30^), primary care clinic cessation support using physician education, support staff, and telephone counseling was compared to providing physicians at four separate clinics with a clinical practice reference, a patient cessation brochure, and Quitline referral reminders. Physicians were educated to incorporate brief advice, pharmacotherapeutic, and support services information into clinical practice. Self-reported 7-day PPA at 3 months was higher for intervention with telephone counseling than control condition (reference materials) (24.2% vs. 15.7%). Cotinine-verified 7-day PPA was lower at 3 months in both intervention and control conditions (11.0% vs. 3.5%), with more control than intervention smokers receiving pharmacotherapy (30.0% vs. 56.0%).

In a nurse-managed tailored messaging systems intervention with pregnant smokers (Britton et al.^29^), prenatal usual care (recording and verifying smoking) was compared to tailored health messaging with quit readiness and cessation planning. However, at 16 weeks, cotinine-verified CA did not differ between tailored messaging (26.0%) and usual care (34.4%).

#### Long-Term Abstinence

In systems change program at two rural Canadian hospitals (Bottorff et al.^24^), surgical patients who smoked were given multimedia smoking cessation information, and healthcare professionals were provided brief advice training (5A model^49^). This focused on benefits of quitting before surgery, and healthcare professionals shared health benefits brochures with patients. At 12 months, there was no difference in the proportion of smokers advised to quit pre-surgery (56.5% vs. 53.9), or who self-reported reducing or quitting 2 months pre-surgery (47.0% vs. 52.2%). No pre-post program difference was seen in self-reported quitting within 2 months prior to surgery (6.0% vs. 7.8%).

In Bullock et al.,^25^ nurses at 21 health clinics were trained in smoking cessation support for pregnant American women, who were followed from gestation to pregnancy end, and 6 weeks post-end. This educationally focused intervention trained nurse in communication with diverse groups, stress responses, community resources, and social, emotional, and informational support. Usual care was compared to three conditions: Eight weekly mailed booklets (booklets), weekly brief nurse phone calls, and 24-hour beeper access to nurses for social support (support), and weekly social support plus eight booklets (combined). Cotinine-verified smoking status was recorded at baseline. Verified PPA did not differ at delivery (control = 17.2%, booklets = 19.2%, support = 22.0%, and combined = 17.0%) or post-delivery (control = 13.3%, booklets = 13.5%, support = 11.4%, and combined = 12.4%).

Similarly, the Britton et al.^29^ nurse-managed systems change intervention for pregnant smokers that used tailored health messaging found no difference in long-term abstinence at 28 weeks, though found higher abstinence in intervention over control participants at postpartum visit (25.0% vs. 15.6%).

Lastly, Hancock et al.,^31^ low long-term abstinence was also reported in a systems change intervention delivered in 10 rural towns. This intervention provided smoke-free environment promotion, self-help quit materials, workplace education, and health promotion. At 3 years post-baseline, self-reported abstinence was no higher in the intervention (20.4%) versus nonintervention (16.9%) towns.

## Discussion

This review identifies several effective smoking cessation intervention approaches for RRR smokers in supporting short-term (<6 months) and long-term (≥6 months) smoking abstinence. Findings from this review suggested that intervention components that work well with RRR populations are broadly consistent with those most effective with general populations. However, as “rurality” captures populations with very different characteristics (eg, pregnant women, Appalachian adolescents), interventions require careful tailoring and co-design to best support long-term smoking abstinence outcomes.

Contingency, incentive, and online smoking cessation approaches are most effective in promoting short-term verified abstinence among RRR smokers, and can exceed phone counseling in effectiveness.^44^ This is consistent with review findings that reward-focused cessation interventions more strongly predict cessation in general, non-rural, populations.^50^

Additionally, as abstinence decreases after contingencies end,^28,34,39^ participant retention is an essential consideration for online intervention success.^28,34,39,44^ Whilst we found incentives to be effective only in the short term, a recent Cochrane review supports their effectiveness for long-term cessation.^51^ However, this evidence is broader than RRR populations, suggesting there may be a difference in contextual elements unique to RRR populations and indicating the need for further exploration into factors such as the level of incentive, and potential role of self-incentive (eg, deposit) in different settings. Improving the usability of online interfaces is another way to support effectiveness.^28^ As abstinence can decrease more rapidly in online versus telephone-only interventions,^44^ trade-offs in benefits should be considered in intervention design (eg, accessibility). Behavior-change program interfaces are a key means of tailoring interventions for RRR populations to account for different needs (eg, real-time input/monitoring).^52^

Telehealth interventions produced moderately low short-term abstinence that was comparable to in-person support.^18^ This is ideal for RRR people who smoke, where in-person services may be less easily accessed, as phone counseling for smoking cessation can be provided within systems change interventions.^30^ Yet, telehealth suitability for long-term outcomes in such populations was less supported, with low abstinence,^43^ issues sustaining abstinence,^25^ and potential cost barriers (eg, integrated counseling).^27^ Despite this, online and telehealth-based interventions are highly suited to RRR delivery as their design account for environmental factors (eg distance), participation requirements (eg, digital literacy), and incorporate pharmacotherapy^26^ (eg, via mail-out NRT).

Pharmacotherapy paired with behavioral support is considered a first-line cessation treatment option.^53^ Yet, combining psychological and pharmacological approaches to cessation with rural populations has mixed support, as community-delivered and financially incentivised^34^ approaches produced similar outcomes to NRT with psychological support.^47^ As short-term abstinence may also not differ based on rurality for such combined programs,^36^ research is needed to align these to RRR-specific needs. Despite brief CBT and pharmacotherapy advice being cost-effective,^32^ there may be low benefit beyond 12 months, to provide psychological support additional to NRT.^26,36,43^ More intensive CBT paired with NRT may, however, help sustain abstinence long-term.^47^ A further alternative that has not yet been tested is the role of electronic cigarettes in RRR adult smoking cessation,^54^ a current research gap.

Training RRR health providers in smoking cessation support holds promise for short-term, though not long-term, abstinence.^46,47^ Although this approach readily integrates into clinical care, providers may not advise smokers to quit,^24^ instead offering printed resources to support long-term abstinence.^25^ A key benefit is that this approach facilitates Aboriginal-led cessation support for long-term abstinence.^38,46^

Educational interventions may support comparable short-term abstinence to other approaches,^18,29^ though this is lower when verified.^33,36^ Despite this, such interventions are readily delivered in clinical settings,^30,36^ and can benefit from considering gender,^31,33^ pregnancy,^29,35^ and cultural^42^ factors. However, long-term abstinence was not well supported for educational interventions (7.8% to 28%).^18,24,25,31,32,35,37^ Given this, it will be worthwhile to consider how engagement with smoking cessation and abstinence education can be sustained and scaled over time for RRR populations specifically.

Using pharmacotherapy alone had mixed evidence, as this was typically combined with other intervention components. However, pharmacotherapy access can support short-term smoking abstinence with behavior modification,^36^ and by using buproprion alone.^48^ Long-term abstinence was not clearly supported for pharmacotherapy, though this may reduce the amount of smoking.^45^ Using pharmacotherapy alone did not produce differing abstinence to interventions that paired this with psychological components (eg, CBT^26,43^). Abstinence effects can subside long-term,^36,47^ and pharmacotherapy side effects are a barrier to adherence.^45^ To address this, Mersha et al.^55^ noted that physical and mental capability, perceptions of NRT and cessation, motivational and social factors need to be incorporated into NRT approaches. This may extend to other forms of pharmacotherapy.

### Future Directions

Registering evaluation protocols is needed to better understand which RRR populations are most suited to telehealth interventions, core needs and intervention tailoring for smoking cessation support, how to design intervention components for these needs, and how this impacts standardized smoking abstinence outcomes. Unique contribution of intervention components to abstinence outcomes can then be isolated (eg, systems interventions),^56^ supporting evidence consolidation for RRR program and policy design. Interventions showing promise include those using pharmacotherapy for short-term abstinence, particularly finding cost-effective ways to address retention, adherence, and scale-up barriers. Research into approaches such as pharmacotherapy (eg, NRT, buproprion, varenicline) is needed, as its delivery can promote relapse prevention, while meeting RRR smokers’ access barriers.^57^ Interventions should also consider the relative benefits of pharmacotherapy advice only versus pharmacotherapy provision, as some RRR populations may have barriers to, for example, NRT access based on advice (eg, relative social disadvantage). There is a need to identify how to maintain smoking abstinence beyond 6 months, by identifying RRR smoker intervention preferences,^40^ and characteristics of former smokers maintaining abstinence long-term. Digital literacy and technology needs are key RRR telehealth considerations.^58^

### Limitations

Although the search strategy captured a range of interventions, heterogeneity in design, content, and abstinence outcome measurement shows the need for standardized evaluation protocols. For example, there could be more use of biochemical verification of smoking abstinence. For example,^59^ Few studies provided NRT to smokers,^60,61^ or used motivational interviewing, for example,^26,27,35,43^ or CBT, for example,^27,32,47^ to improve outcomes.^62^ As only one telehealth study was post-2016,^34^ more modern RRR telehealth studies will be included in further reviews. Systems interventions, unspecified pharmacotherapy dosage,^28,45^ and small samples^48^ complicate interpretation of abstinence outcomes. Despite these limitations, this study provides a detailed overview of how smoking cessation interventions can be best delivered to RRR populations.

## Conclusions

Our review found that most interventions have limited short-term influence on RRR smoking abstinence (<50.0%), with this decreasing markedly beyond the 6-month mark. Interventions for RRR smokers should include ­pharmacotherapy paired with psychological smoking cessation counseling (eg, CBT) per recommendations,^11^ to establish and maintain short-term abstinence. Interventions using contingency designs that monitor and reward RRR smokers’ intervention adherence are best suited to supporting short-term abstinence. Designing follow-up mechanisms is critical to supporting RRR people in their smoking abstinence beyond 6 months in different intervention settings, including but not limited to the home, the workplace, clinical, and community settings. This is the next step in helping RRR-living people to stay smoke-free after quitting.

## Supplementary Material

A Contributorship Form detailing each author’s specific involvement with this content, as well as any supplementary data, are available online at https://academic.oup.com/ntr.

ntad098_suppl_Supplementary_Materials

## Data Availability

Data are available in supplementary material, and in original cited works.
