# Supplementary material for: Smoking Cessation Interventions and Abstinence Outcomes for People Living in Rural, Regional, and Remote Areas of Three High-Income Countries: A Systematic Review
Source: Nicotine Tob Res. 2023 Jun 20;25(11):1709–18. doi: 10.1093/ntr/ntad098 (PMC10475608; doi:10.1093/ntr/ntad098)
Supplement: ntad098_suppl_Supplementary_Materials [file ntad098_suppl_supplementary_materials.zip › R2_RRRSCI_Supplement_1_search_strategy_060423_FINAL.docx]

**Supplementary File 1**

**Systematic Database Search Strategy**

Ovid MEDLINE (epub ahead of print, in-process & other non-indexed citations and daily)

(Searched: 28/02/2021, updated: 29/06/2022)

| # | Query | Results |
| --- | --- | --- |
| 1 | smoking cessation.mp. or Smoking Cessation/ | 38187 |
| 2 | "TOBACCO USE CESSATION".mp. or "Tobacco Use Cessation"/ | 3046 |
| 3 | ((ceas* or cess* or prevent* or stop* or quit* or abstin* or abstain* or reduc*) adj5 (smok* or tobacco or nicotine)).mp. [mp=title, abstract, original title, name of substance word, subject heading word, floating sub-heading word, keyword heading word, organism supplementary concept word, protocol supplementary concept word, rare disease supplementary concept word, unique identifier, synonyms] | 70922 |
| 4 | 1 or 2 or 3 | 70922 |
| 5 | rural.mp. or Rural Population/ | 163306 |
| 6 | remote.mp. | 73333 |
| 7 | isolated.mp. | 954622 |
| 8 | 5 or 6 or 7 | 1179138 |
| 9 | (intervention* or trial* or pilot* or program*).mp. [mp=title, abstract, original title, name of substance word, subject heading word, floating sub-heading word, keyword heading word, organism supplementary concept word, protocol supplementary concept word, rare disease supplementary concept word, unique identifier, synonyms] | 3347862 |
| 10 | (australia* or canada* or america* or united states).mp. [mp=title, abstract, original title, name of substance word, subject heading word, floating sub-heading word, keyword heading word, organism supplementary concept word, protocol supplementary concept word, rare disease supplementary concept word, unique identifier, synonyms] | 1641314 |
| 11 | usa.mp. | 100043 |
| 12 | 10 or 11 | 1711715 |
| 13 | 4 and 8 and 9 and 12 | 207 |
| 14 | 4 and 8 and 9 | 823 |

CINAHL (Searched: 28/02/2021 updated: 29/06/2022)

| # | Query | Results |
| --- | --- | --- |
| 1 | (MH "Smoking Cessation") OR "smoking cessation" | 28009 |
| 2 | "tobacco use cessation" | 521 |
| 3 | ((ceas* or cess* or prevent* or stop* or quit* or abstin* or abstain* or reduc*) n5 (smok* or tobacco or nicotine)) | 42287 |
| 4 | S1 OR S2 OR S3 | 42287 |
| 5 | (MH "Rural Population") OR (MH "Rural Areas") OR "rural" | 75131 |
| 6 | "remote" | 17831 |
| 7 | "isolated" | 65480 |
| 8 | S5 OR S6 OR S7 | 152445 |
| 9 | (intervention* or trial* or pilot* or program*) | 1356759 |
| 10 | (australia* or canada* or america* or united states or usa) | 973845 |
| 11 | S4 AND S8 AND S9 AND S10 | 188 |

SCOPUS (Searched: 28/02/2021, updated: 29/06/2022)

| # | Query | Results |
| --- | --- | --- |
| 1 | (TITLE-ABS-KEY ( ( ( ceas* OR cess* OR prevent* OR stop* OR quit* OR abstin* OR abstain* OR reduc* ) W/5 ( smok* OR tobacco OR nicotine ) ) ) AND TITLE-ABS-KEY ( rural OR remote OR isolated ) AND TITLE-ABS-KEY ( ( intervention* OR trial* OR pilot* OR program* ) ) AND TITLE-ABS-KEY ( ( australia* OR canada* OR america* OR united AND states OR usa ) ) ) | 225 |

EMBASE (Searched: 1947-28/02/2021, updated: 29/06/2022)

| # | Query | Results |
| --- | --- | --- |
| 1 | smoking cessation.mp. or Smoking Cessation/ | 65059 |
| 2 | "TOBACCO USE CESSATION".mp. or "Tobacco Use Cessation"/ | 58412 |
| 3 | ((ceas* or cess* or prevent* or stop* or quit* or abstin* or abstain* or reduc*) adj5 (smok* or tobacco or nicotine)).mp. [mp=title, abstract, heading word, drug trade name, original title, device manufacturer, drug manufacturer, device trade name, keyword, floating subheading word, candidate term word] | 96670 |
| 4 | 1 or 2 or 3 | 96670 |
| 5 | rural.mp. or Rural Population/ | 198004 |
| 6 | remote.mp. | 92147 |
| 7 | isolated.mp. | 1239912 |
| 8 | 5 or 6 or 7 | 1515201 |
| 9 | (intervention* or trial* or pilot* or program*).mp. [mp=title, abstract, heading word, drug trade name, original title, device manufacturer, drug manufacturer, device trade name, keyword, floating subheading word, candidate term word] | 4901585 |
| 10 | (australia* or canada* or america* or united states).mp. [mp=title, abstract, heading word, drug trade name, original title, device manufacturer, drug manufacturer, device trade name, keyword, floating subheading word, candidate term word] | 3723044 |
| 11 | usa.mp. | 213432 |
| 12 | 10 or 11 | 3863453 |
| 13 | 4 and 8 and 9 and 12 | 389 |
| 14 | 4 and 8 and 9 | 1130 |

INFORMIT HEALTH (Searched: 28/02/2021, updated: 29/06/2022)

| # | Query | Results |
| --- | --- | --- |
| 1 | ((ceas* OR cess* OR prevent* OR stop* OR quit* OR abstin* OR abstain* OR reduc*) %5 (smok* OR tobacco OR nicotine)) AND  (rural OR remote OR isolated) AND  (intervention* OR trial* OR pilot* OR program*) AND  (australia* OR canada* OR america* OR united states OR usa) | 163 |

COCHRANE LIBRARY (Searched: 28/02/2021, updated: 29/06/2022)

| # | Query | Results |
| --- | --- | --- |
| 1 | (ceas* OR cess* OR prevent* OR stop* OR quit* OR abstin* OR abstain* OR reduc*) AND (smok* OR tobacco OR nicotine) AND (rural OR remote OR isolated) AND (intervention* OR trial* OR pilot* OR program*) AND (australia* OR canada* OR america* OR united states OR usa) | 150 |

PSYCHINFO (Searched 1806 to 28/02/2021, updated: 29/06/2022)

| # | Query | Results |
| --- | --- | --- |
| 1 | smoking cessation.mp. or Smoking Cessation/ | 17771 |
| 2 | "TOBACCO USE CESSATION".mp. or "Tobacco Use Cessation"/ | 344 |
| 3 | ((ceas* or cess* or prevent* or stop* or quit* or abstin* or abstain* or reduc*) adj5 (smok* or tobacco or nicotine)).mp. [mp=title, abstract, heading word, table of contents, key concepts, original title, tests & measures, mesh] | 24506 |
| 4 | 1 or 2 or 3 | 25405 |
| 5 | rural.mp. or Rural Population/ | 44673 |
| 6 | remote.mp. | 11240 |
| 7 | isolated.mp. | 30351 |
| 8 | 5 or 6 or 7 | 83973 |
| 9 | (intervention* or trial* or pilot* or program*).mp. [mp=title, abstract, heading word, table of contents, key concepts, original title, tests & measures, mesh] | 885484 |
| 10 | (australia* or canada* or america* or united states).mp. [mp=title, abstract, heading word, table of contents, key concepts, original title, tests & measures, mesh] | 420118 |
| 11 | usa.mp. | 16644 |
| 12 | 10 or 11 | 430382 |
| 13 | 4 and 8 and 9 and 12 | 92 |
| 14 | 4 and 8 and 9 | 273 |
|  | Total results | 1414 |
