# Supplementary material for: Smoking Cessation Interventions and Abstinence Outcomes for People Living in Rural, Regional, and Remote Areas of Three High-Income Countries: A Systematic Review
Source: Nicotine Tob Res. 2023 Jun 20;25(11):1709–18. doi: 10.1093/ntr/ntad098 (PMC10475608; doi:10.1093/ntr/ntad098)
Supplement: ntad098_suppl_Supplementary_Materials [file ntad098_suppl_supplementary_materials.zip › R2_RRRSCI_Supplement_2_study_details_060423_FINAL.docx]

| **Supplement 2.** Tobacco smoking cessation intervention study characteristics (N=26). | | | | |
| --- | --- | --- | --- | --- |
| Study | Design/population | Intervention | Abstinence: <6 months | Abstinence: ≥6 months |
| Adams  2006 | Pre-post; rural Victorian Aboriginal Australian smokers (n=32); gender: NR; age: NR; duration=3 weeks; | Aboriginal health service delivered educational, resource, and Quitline support, with NRT/bupropion access. No control. | PPA at 3-weeks:19.0% (p=NR) | — |
| Azor Hui  2013 | Prospective follow-up (randomised); rural smokers in primary care (n=333); female (62.2%); age: NR; duration=24 months; USA | Moderate-intensity management: 2 telephone counselling sessions every 6 months. High-intensity management: pharmacotherapy with ≤6 quit counselling calls, and pharmacotherapy (nicotine patch/bupropion). Pharmacological management: quit education materials and offer of a 6-week nicotine patch or a 7-week bupropion course. | — | 7-day PPA at 36 months: 3.7-4.3% (p<.05); comparator=5.3% |
| Bailey  2015 | RCT; rural pregnant women (n=1947); age (mean): intervention=24.2; control=24.5; duration=first antenatal visit to delivery; USA. | Four 15–30-minute sessions of psychoeducation, brief intervention, and motivational interviewing to quit smoking. Usual care control: brief advice to quit, quit referral and follow-up. | — | Complete abstinence: 28.1% (p<.001); control=9.8% |
| Bottorff  2016 | Between-subjects; rural Canadian smokers (n=240); Female=59.3%; age (mean): 51.7 years; duration=NR | This included education in the 5As, online training, and resources for healthcare providers (e.g., posters for common areas). Participants received brochures to encourage patients to abstain after surgery. | — | Self-report quit 1 year post intervention: 7.8% (p=.432); comparator= 6.0% |
| Breen 2021 | Prospective follow-up; rural Tasmanian smokers (n=62); female (66.1%); age (mean): 47.9; duration: 3 months. | Pharmacy program of 7x15-minute sessions, including 6 check-ins with brief cessation advice and telephone support offering, with incentives: enrolment ($10.00, abstinence at check-ins ($50.00). No control. | — | 7-day PPA verified, week 1 (32.3%), month 2 (21.0%), month 3 (19.4%) |
| Britton  2006 | Quasi-experimental; pregnant women (n=194); age (mean/): control (23.0); experimental (24.4); duration: approx. 28 weeks; USA | Systems change intervention of tailored health messaging on quitting, quit readiness assessment and planning, and a cessation tips calendar for quit date, prenatal care appointments and pregnancy milestones. Usual care control participants received prenatal care per routine protocol. | Self-report verified CA at 16 weeks: 26.0% (p=NR); comparator=34.4% | Self-report verified CA at 28 weeks: 25.0% (p=.017); comparator=15.6% |
| Bullock  2009 | RCT; pregnant women (n=695) in nutritional clinics; age (mean): treatments (23.1); control (23.9); duration: approx. 34 weeks; USA | Systems change intervention with nurses trained in telephone social support, resources knowledge for participants, with 4 conditions: support plus information booklets; support only; information booklets alone; control (no intervention). Support included weekly calls and 24-hour nurse access, with smoking a secondary focus. Eight booklets were provided, one per week. Control condition was no intervention. | — | ITT PPA: support (11.4%), booklets (12.4%), support plus booklets (13.5%) (p=.710); control=17.2% |
| Byaruh-anga  2021 | RCT; rural smokers (n=655): female=-77.4%; age (mean): 43.2-44.2; duration= 6 weeks/4-month follow-up; Australia | Participants were randomised to three groups: video counselling: (≤ six 15-minute video sessions with a quit advisor (week 1, days 3, 7, 14, 30), or 2, 4, and 6-weeks later if no quit date set. CBT/MI sessions covered triggers, prevention, self-efficacy, and pharmacotherapy advice only); telephone counselling (same approach as the video counselling, via telephone) or control (written materials on quitting support and aids). | 7-day PPA 4-months post baseline: video 18.9% (p=.003); phone 12.7% (NR); 3-month CA: video 7.0% (p=.07); phone 4.4% (p=NR); control=8.9% | — |
| Carlson  2012 | Prospective follow-up; rural smokers (n=554); in 16 telehealth sites in Canada; Female= 65.6%-72.8%; age (mean): 47.2-47.3; duration =16 weeks | Eight 90-minute sessions with clinical psychologists: 1-5 were weekly, with a group quit night after session three, and remaining sessions spaced over 8 weeks. These covered self-monitoring of smoking, restricting smoking, nicotine fading, cessation aids, withdrawal, weight, stress, relapse management, and group support. No control. | ITT 3-month CA: 3 months: 25.5% (p=.660) | ITT 3-month CA: 6 (14.1%, p=.840), 12 months (25.5%, p=.240) |
| Ellerbeck  2009 | RCT single blind; rural smokers (n=750) in primary care practices; female (58.5%); age (mean): 42.7; duration=24 months; USA | See Cox et al. (2008) design, described in Azor Hui et al. (2013): Moderate-intensity, high-intensity, and pharmacological management (comparator). | — | Verified 7-day PPA at 24 months: MDM (14.7%, p=NR); HDM (14.8%, p=NR); PM (13.5%, p=NR). |
| Ferketich  2014 | Cluster-RCT; Appalachian residents (n=214) at primary care clinics and via phone; Female=60-79%; age (mean): treatment=37.6; control= 47.5; duration=12 weeks; USA | Systems change intervention including a physician education session on the 5As, discussion on brief counselling in clinical practice, pharmacotherapy and Medicaid information, and cessation handouts. Physicians received a comparison of their counselling to other physicians. Clinics had a dedicated employee prioritising cessation, and quit counselling was available to support patients. In control clinics, physicians received a quick reference guide on the 5As, pharmacotherapy, and Medicaid, and smokers received a quitline brochure and pharmacotherapy handout on Medicaid. | 7-day PPA at 3-months: self-report (24.2%, p<.05), verified (11.0%, p<.05); control: self-report=15.7%, verified=3.5% | — |
| Gould  2015 | Pre-post repeated measures; rural smokers in primary care (n=42); Female=57.0%; age: 25-34(9.0%), 35-44(31.0%), 45-54(33.0%), 55-64(19.0%), >65(7.0%); duration=2 hours/6-month follow-up; Australia | Single 2-hour session: educational and group interactive work on behavioural change techniques in CBT for smoking cessation (i.e., smoking and quitting reasons, nicotine, and pharmacotherapy actions), limited samples of nicotine patches, gum/lozenges (e.g., 1-2 patches), quit, relapse prevention, and support planning. No control. | — | Self-report quit at 6-months: 28.6% (p=NR); verified (14.3%, p=NR) |
| Hancock  2001 | RCT; Residents of 20 rural Australian towns (n=10256); baseline: control, n=1103; intervention, n=1280); gender=NR; age (range): 18-70; duration: intervention=NR; Australia | Systems change intervention for implementing smoke-free environments in workplaces, restaurants, hotels, motels, sporting clubs, community clubs, and eating places, providing quit smoking self-help materials, programs, and passive smoking health and legal education to employees. | — | Self-report quit=20.4% (p>.05); control=16.9% |
| Harris  2015 | RCT; rural Appalachian pregnant smokers (n=17); age (mean): CM, 24.0; SCHB, 24.2; duration=approx. 6-weeks/2 follow-ups; USA | Two programs were used: intensive web-based contingency management (CM), where participants received a 6-week web-based CM program with two follow-up sessions after the program but before birth, and smoking cessation for healthy births (SCHB), providing five calls with a counsellor during pregnancy. Cessation measured at months 1(T1); 2(T2); 3(T3); CM: 6.9(T4), 8.1(T5), 8.8(T6); SCHB: 6.5(T4), 7.4(T5), 8.2(T6). No control. | Verified self-report CA (CM/SCHB): T1 (14.3/10.0%), T2 (28.6/20.0%), T3 (28.6/20.0%), (ps=NR) | Verified self-report CA: (CM/SCHB): T4 (28.6/30.0%), T5 (14.3/30.0%), T6 (14.3/30.0%). |
| Horn  2004 | Cluster-matched RCT; rural teens (n=258; NOT, n=124; BI, n=134) at 20 public high schools in rural Virginia/North Carolina; female=56.2%; age (mean): NOT, 16.2; BI, 16.3; duration=approx. 3 months; USA | The not on tobacco (NOT) intervention delivered weekly 10-hr-long sessions in same-gender groups, with a same-gender facilitator. Teens received support for understanding reasons for smoking, quit preparation, nicotine addiction, social support, and managing stress, and relapse. The comparator was mixed-gender groups receiving a single, 15-min classroom brief intervention (BI) of quit smoking advice and self-help brochures. No control. | Verified 7/30-day PPA at 12-weeks: NOT=8.3%, BI=1.7% (p=.030) | — |
| Ivers  2003 | Pre-post-test; Aboriginal and Torres Strait Islander Australians (n=111) at an Aboriginal Health Service; Female: intervention=60%; control=38%; age (≤30, >30 years): intervention (33%, 67%); control (61%, 39%); duration=6 weeks; Australia | Brief 5-minute intervention (BI) advice on quitting, health effects, quit support, and quit readiness counselling), a chart and pamphlet on tobacco. Participants were provided a weekly supplies of nicotine patches (6-weeks/21mg, 2-weeks/14mg, 2-weeks/7mg patches) and could request more. No control. | — | Verified 7/30-day PPA at 6 months: BI: 10.0% (p=NR); NRT-BI=3.0% (p=NR). |
| Marley  2014 | RCT; Aboriginal and Torres Strait Islander Australians (n=168) at 2 Aboriginal Health Services in two remote towns in Kimberley, Australia; female: intervention=64%, usual care=50%; age (mean): intervention, 41.9; usual care, 38.3; duration=12 months; Australia | In-person counselling (weeks 1-4 weekly, then monthly to 6-months, then one every two months, covering motivational interviewing, smoking triggers, relapses, and weight, health benefits, pharmacotherapy referral, smoking motivators, connection with support agencies (e.g., housing). Pharmacotherapy was accessible, but not elaborated. Comparator participants received quit advice, self-initiated follow up, and pharmacotherapy (dosage unspecified). | — | Intervention/control: Self-report 7-day PPA at 6-months: (9.0%/ 11.0%, p=NR); Verified 7-day PPA at 12-months CC: (29.0%/8.0%, p=.009); Self-report 7-day PPA at 12-months ITT: (11.0%/5.0%, p=.131) |
| Northridge  2008 | Pre-post-test; Appalachian adults (n=725) in 5 counties in rural West Virginia; female=71.5%; age=NR; duration=approx. 8 weeks; USA | Intervention included medical examination, an 8-session behaviour change program modelled on Freedom from Smoking (American Lung Association) with follow-up support group meetings, and an 8-week free supply of bupropion or nicotine replacement therapy (NRT). No control condition. | Self-report quit at 2-months: rural (51.4%) (p=NR), urban (60.6%) (p=NR); random sample (75.0%) (p=NR) | Self-report quit at 12-months: random sample (17.0%) (p=NR) |
| Reynolds  2015 | RCT; rural Appalachian non-treatment seeking adolescents (n=62); female: AT=48.4%, CT=51.6%; duration=6 weeks; USA | Home-based 6-week contingency management active treatment (AT), providing vouchers for 3 daily webcam confirmed breath samples verifying abstinence (CO<4ppm). Vouchers were staggered: days 1-7, $6/day; days 8-12, $3/sample; days 13-21, $3 for first sample meeting criterion, increasing by $0.25 increments for subsequent tests, then $5 for every five consecutive criteria samples; days 22-27, $6/day; days 28-32, $6/day for 3 timely samples, with no CO criterion. Control condition (CT), participants received vouchers for timely breath samples with no CO verification, in the same staggered values as AT. | AT/CT: mean self-report cigarettes p/day, last 14-days: baseline: (11.3/11.8, p>.05); abstinence days 12-32:(NR/NR). CO ppm: baseline: (NR/NR); abstinence: (4.7/9.5, p<.01). | — |
| Richter  2015 | RCT, 2-arm randomised; Kansas primary care patients (n=566); female, ITM=62.7%, phone=66.9%; age (mean): ITM=47.3, phone=47.5; duration=8 weeks; USA | Integrated telemedicine counselling (ITM) using real-time clinic webcam sessions. Control participants received 4 telephone counselling (phone) sessions. If participants created a quit plan and/or desired pharmacotherapy, the plan and a prescription request form were mailed to participant, to arrange with their health care provider. Duration, content, and pharmacotherapy support were identical in both groups. | — | Verified 7-day PPA at 12-months (ITT): ITM=12.0%, phone=9.8% (p=.406) |
| Santiago-Torres  2022 | RCT, 2-arm; rural US residents (n=550); male: QG=23.0%, iCQ=28.0%; age (mean)=38.4; duration= self-paced access to sequential content; USA | An acceptance and commitment therapy smartphone application iCanQuit (iCQ) was compared to a US Clinical Practice Guidelines app QuitGuide (QG) for smoking cessation. iCanQuit taught skills to manage smoking urges, motivation, and relapse in 8 levels with exercises to plan quitting. This included tobacco treatment, smoking trigger, and smoke-free skills, and was not rural tailored. Comparator participants used QuitGuide to increase quit motivation via reasoning and health information, identify smoking triggers, supports and smoke-free benefits. | iCQ/QG: 7-day PPA at 3 months (26%/ 17%, p=.012), 30-day PPA at 3 months (15%/9%, p=.041) | iCQ/QG: 7-day PPA at 6 months: 35%/ 27%, p=.081; 7-day PPA at 12 months: 35%/31%, p=.308; 30-day PPA at 12 months: 25%/18%, p=.089; 30-day PPA at 12 months: 29%/ 25%, p=.391; CA: 15%/10%, p=.105 |
| Schorling  1997 | Cluster RCT; African American daily smokers in rural Virginia (n=896); 70 Louisiana (LC)/Buckingham county (BC) clinics; male: LC=56.3%, BC=53.5%; age (mean): LC=41.5, BC=40.1; duration=18 months; USA | Treatment participants (BC) received smoking cessation interventions designed with a church coalition, providing one-on-one counselling with self-help materials. Counselors were trained in the stages of change model and providing self-help materials (e.g., smoking cessation goals calendar). Control participants (LC) received hypertension, dietary and exercise counselling with no smoking cessation support. | — | 30-day PPA CA at 18-months: BC=9.6%, LC=6.2%, p>.05 |
| Sheffer  2004 | Pre-post-test; low-SES primary care patients (n=1644) in 15 Arkansan clinics and hospitals; female=67.0%; age=NR; duration=NR; USA | State-wide tobacco control program tobacco treatment specialists (TTS) were employed at each participating study site. Specialists delivered intensive cessation treatment (group and individual) sessions (model unspecified). Outcomes compared to all patients. | Self-report CC quit: post treatment and program: TTS= 74.0%, all= 42%, p=NR; 3 months post discharge (TTS= 42.0%, all=30.0%, p=NR) | — |
| Sheffer  2009 | Pre-post-test; rural Arkansan residents (n=2350); 20 community clinic/hospital sites; female=68.0%; age (mean): 46.1; duration=6 weeks; USA | Intensive multi-component intervention using CBT with relapse prevention delivered by tobacco treatment specialists over six 60-minute group or individual sessions, with nicotine patches available (dosage unspecified). No control. | 7-day PPA CA 3 months post complete: CC=28.6%/ITT=19.0% (ps=NR) | 7-day PPA CA at 12-months: CC=24.3%/ITT=13.0% (ps=NR) |
| Stoops  2009 | RCT; Appalachian residents (n=68) in 54 Kentucky counties; female: AC=74.3%, YC=75.8%; age (mean): AC=38.0, YC=40; Duration=6 weeks; USA | Abstinence contingency (AC) condition participants (n=35) recorded 2 daily carbon monoxide sample videos and readings via online software that gave feedback and reinforcement based on smoking status. AC participants received financial incentives contingent on verified smoking abstinence. Yoked control (YC) participants (n=33) received monetary incentives independent of smoking with reinforcement matched to AC participants' schedule, set a quit date, and were told reinforcement would be random after video upload. | ITT verified abstinence (AC/YC): W1 (30.4%/ 18.4%, p<.05); W2 (39.0%/ 8.7%, p<.05); W3 (39.8%/ 6.7%, p<.05); W4 (36.9%/ 10.0%, p<.05); W5 (39.8%/ 11.3%, p<NR); W6 (33.9%/ 12.6%, p<NR) | — |
| Wong  2004 | Pre-post-test; rural primary care patients (n=18), Tamworth, Australia; female=66.6%; age (mean)=49; duration=8 weeks; Australia | Prescription bupropion SR was provided for 8-weeks. | Self-report PPA CC at 2- and 3-months post completion: 2-month=38.9%, 3-month=38.9% | — |

**Note:** NR: not reported; ITT: intention to treat; CC: complete case; CA: complete abstinence; verified=biochemically verified (see Supplement 2 for details); PPM: parts per million.
