# Supplementary material for: Smoking Cessation Interventions and Abstinence Outcomes for People Living in Rural, Regional, and Remote Areas of Three High-Income Countries: A Systematic Review
Source: Nicotine Tob Res. 2023 Jun 20;25(11):1709–18. doi: 10.1093/ntr/ntad098 (PMC10475608; doi:10.1093/ntr/ntad098)
Supplement: ntad098_suppl_Supplementary_Materials [file ntad098_suppl_supplementary_materials.zip › R2_RRRSCI_Supplement_3_intervention_outcomes_060423_FINAL.docx]

**Supplementary File 3**

| **Table.** Summary of follow-up smoking cessation outcome measures and abstinence rates for included studies. | | | | | | | | |
| --- | --- | --- | --- | --- | --- | --- | --- | --- |
|  | Sample size | | Abstinence measure | Percent abstinent <6 months | | Abstinence measure | Percent abstinent ≥6 months | |
| Author  Year  Country | Control/  comparator | Intervention | <6 months | Control/  comparator | Intervention  [significance] | ≥6 months | Control/  comparator | Intervention  [significance] |
| Adams  2006  Australia[^1^](#_ENREF_1) | - | n=32 | PPA at 3-weeks | - | 19% (n=6)  [p=NR] | - | - | - |
| Azor Hui  2013  USA[^2^](#_ENREF_2) | PM (n=131) | LDM (n=109)  HDM (n=93) | - | - | - | 7-day PPA at 36 months | 5.3% (n=7)  [referent] | LDM (3.7%, n=4) [OR=0.68, p<.05]  HDM (4.3%, n=4) [OR=0.80, p<.05] |
| Bailey  2015  USA[^3^](#_ENREF_3) | n=461 | n=1486 | - | - | - | CA at end of trimester 2 (nonspecific ~13-26 weeks) | 9.8% (n=45) | 28.1% (n=418)  [p_diff_<.001] |
| Bottorff 2016 Canada[^4^](#_ENREF_4) | - | Pre-SSBS (n=240)  Post-SSBS (n=90) | - | - | - | Self -report as ‘quit’ within 2-months pre-surgery, at 1 year post intervention. | - | Pre-SBBS, CC: 6.0% (n=9/150)  Post-SBBS, CC: 7.8% (n=7/90)  [p_diff_=.432] |
| Breen  2021  Australia[^5^](#_ENREF_5) | - | n=62 | Biochemically verified (CO) [7-day PPA/CA] at weeks 1-4, months 2, 3. | - | Week 1: 32.3% (n=17)  Month 2: 21.0% (n=13)  Month 3: 19.4% (n=12)  [p=NR] | - | - | - |
| Britton  2006  USA[^6^](#_ENREF_6) | n=93 | n=101 | Self-report CA at 16 weeks, and cotinine verified (urinary <200ng/ml) at 16-weeks | 44.3% CA,  34.4% cotinine (-) | 34.2% CA [p=NR],  26.0% cotinine (-) [p=NR] | Self-report CA at 28-weeks and at postpartum, and cotinine verified (urinary <200ng/ml) at 28-weeks and postpartum. | 49.2% CA, 31.3% cotinine (-)  16.7% CA,  15.6% cotinine (-) | 45.2% CA,  29.1% cotinine (-)  37.3% CA [p_diff_=.037],  25.0% cotinine (-) [p_diff_=.017] |
| Bullock  2009  USA[^7^](#_ENREF_7) | n=171 | Social support (SSB) and booklets (n=170)  Social support alone (SSA) (n=175)  Booklets alone (BA) (n=171) | - | - | - | Biochemically verified ITT PPA (salivary cotinine≤30ng/ml) at late pregnancy (time unspecified) and 6-weeks post-partum. | Late pregnancy, 17.2% (n=22);  Post-delivery, 13.3%(n=17) | Late pregnancy [χ^2^(3)=1.33, p=.720]:  SSB, 17.0% (n=22);  SSA, 22.0% (n=29);  BA, 19.2% (n=27).  Post-delivery [χ^2^(3)=1.39, p=.710]:  SSB, 12.4% (n=16);  SSA, 11.4% (n=15);  BA, 13.5% (n=19). |
| Byaruhanga  2021  Australia[^8^](#_ENREF_8) | n=225 | Video  (n=201)  Telephone (n=229) | 7-day PPA at 4-months post baseline; 3-month CA | 8.9% (n=20) PPA  [referent] | Video, 18.9%(n=38) PPA [p=.003]  Telephone, 12.7%(n=29) PPA [NR];  Video, 7.0%(n=14) CA [p=.07]  Telephone, 4.4%(n=10) CA  [NR] | - | - | - |
| Carlson  2012  Canada[^9^](#_ENREF_9) | Tom Baker Cancer Centre (TBCC) (n=370) | Telehealth  (TH)  (n=184) | 3-month CA at 3 months | ITT:  3 months 27.3% (n=101) | ITT:  3 months 25.5% (n=47) [χ^2^(1)=0.19, p=.660] | 3-month CA at 6 and 12 months | ITT: 6 months 13.5% (n=50); 12 months 21.1% (n=78) | ITT: 6 months 14.1% (n=26) [χ^2^(1)=0.04, p=.840]; 12 months 25.5% (n=47) [χ^2^(1)=1.40, p=.240] |
| Ellerbeck  2009  USA[^10^](#_ENREF_10) | Pharmacotherapy management (PM) (n=250) | Moderate-intensity disease management (MDM) (n=249) and high-intensity disease management (HDM) (n=251) | - | - | - | Self-reported 7-day PPA at 24 months; and biochemically verified (salivary cotinine <15ng/ml) at 24-months | PM, 23.0% (n=56/244) [referent^*^]; cotinine verified:  PM 13.5% (n=33/244) | MDM, 23.5% (n=56/238) [p=NR];  HDM, 27.9% (n=68/244) [p=NR];  *Combined MDM/HDM [OR=1.33, p<.05]  cotinine verified:  MDM  14.7% (n=35/238) [p=NR];  HDM  14.8% (n=36/244) [p=NR]; |
| Ferketich  2014  USA[^11^](#_ENREF_11) | Control clinic (n=4) participants (n=115) | Intervention clinic (n=4) participants (n=99) | 7-day PPA at 3-months; and biochemically verified (salivary cotinine, 14 ng/ml) at 3-months | 15.7% (n=18/115) [referent]; and 3.5% (n=4/115) [referent] | 24.2% (n=24/99) [OR=1.68, p<.05]; and 11.0% (n=11/99) [OR=3.37, p<.05] | - | - | - |
| Gould  2015  Australia[^12^](#_ENREF_12) | - | n=42 | - | - | Self-reported “quit”; and  biochemically verified (CO) at 6-months | - | - | 28.6% (n=12/42) [p=NR]; and 14.3% (n=6/42) [p=NR] |
| Hancock 2001 Australia[^13^](#_ENREF_13) | Towns (n=10), participants (n=1103 baseline smokers) | Towns (n=10), participants (n=1280 baseline smokers) | - | - | - | Self-reported “quit” | 16.9% | 20.4%  [p>.05] |
| Harris  2015  USA[^14^](#_ENREF_14) | - | CM: n=7  SCHB: n=10 | Biochemically verified (urinary cotinine) [Self-reported CA].  CM at (months):  T1 (3.50)  T2 (4.87)  T3 (5.82)  SCHB at (months)  T1 (3.42)  T2 (4.45)  T3 (5.45) | - | CM:  T1 (14.29%, n=1/7) [p=NA]  T2 (28.57%, n=2/7) [p=NA]  T3 (28.57%, n=2/7) [p=NA]  SCHB:  T1 (10.0%, n=1/10) [p=NA]  T2 (20.0%, n=2/10) [p=NA]  T3 (20.0%, n=2/10) [p=NA] | Biochemically verified (urinary cotinine) [Self-reported CA].  CM at (months):  T4 (6.86)  T5 (8.05)  T6 (8.75)  SCHB at (months)  T4 (6.45)  T5 (7.37)  T6 (8.19) | - | CM:  T4 (28.57%, n=2/7) [p=NA]  T5 (14.29%, n=1/7) [p=NA]  T6 (14.29%, n=1/7) [p=NA]  SCHB:  T4 (30.0%, n=3/10) [p=NA]  T5 (30.0%, n=3/10) [p=NA]  T6 (30.0%, n=3/10) [p=NA] |
| Horn  2004  USA[^15^](#_ENREF_15) | - | NOT (n=124)  BI (n=134) | Biochemically verified (CO) [7/30-day PPA/ CA] at 12-weeks | - | NOT (8.33%)  BI (1.67%)  [p=.030] | - | - | - |
| Ivers  2003  Australia[^16^](#_ENREF_16) | - | BI (n=71)  NRT (n=40) | - | - | - | Biochemically verified (CO) [7/30-day PPA/ CA] at 6-months | - | BI: 10.0% (n=unspecified;  n=14 declined CO; n=2 CO present with condition unspecified) [p=NR]  NRT-BI:3.0% (n=unspecified; n=7 declined CO; n=2 CO present with condition unspecified) [p=NR] |
| Marley  2014  Australia [^17^](#_ENREF_17) | n=108 | n=55 | - | - | - | Self-reported 7-day PPA at 6-months  Cotinine 7-day PPA at 12-months; self-reported 7-day PPA at 12-months | 11.0% (n=7/64)  CC: 8.0% (n=5/64)  ITT: 5.0%  (5/108) | 9.0% (n=3/33) [p=NR]  CC: 29.0% (n=8/28) [p=.009];  ITT: 11.0% (6/55)  [p=.131] |
| Northridge  2008  USA[^18^](#_ENREF_18) | - | n=725 | Self-reported “quit” at 2-months. | - | Random sample: (75.0%, n=46/61^*^; [p=NR]  At program completion (≥8 weeks):  Overall (53.10%, n=385/725^*^) [p=NR]  Rural (51.40%, n=281/547^*^) [p=NR]  Urban (60.60%, n=94/155^*^) [p=NR]  **n*s derived from %) | Self-reported “quit” at 12-months. | - | Random sample: (17.0%, n=10/61^*^) [p=NR] |
| Reynolds  2015  USA[^19^](#_ENREF_19) | CT (n=31) | AT (n=31) | Self-reported cigarettes per day/previous 14-days (mean), and CO at (days):  Baseline (1-7)  Shaping (8-11)  Abstinence (12-32)  Thinning (33-37)  R-baseline (38-42) | Cigarettes per day:  Baseline (11.8)  Shaping (NR^*^)  Abstinence (NR^*^)  Thinning (NR^*^)  R-baseline (8.1)  CO ppm:  Baseline (NR^*^)  Shaping (NR^*^)  Abstinence (9.5)  Thinning (NR^*^)  R-baseline (NR^*^)  ^*^Charted only | Cigarettes per day:  Baseline (11.3) [p>.05]  Shaping (NR^*^) [p=NR]  Abstinence (NR^*^) [p>.05]  Thinning (NR^*^) [p=NR]  R-baseline (6.0) [p<.001]  CO ppm:  Baseline (NR^*^) [p>.05]  Shaping (NR^*^) [p<.05]  Abstinence (4.7) [p<.01]  Thinning (NR^*^) [p<.01]  R-baseline (NR^*^) [p<.05]  ^*^Charted only | - | - | - |
| Richter  2015  USA[^20^](#_ENREF_20) | Phone (n=286) | ITM (n=280) | - | - | - | Biochemically verified 7-day PPA [salivary cotinine <15ng/ml, or CO <10ppm] at 12-months | ITT: 9.8% (n=27/280) | ITT: 12.0% (34/286) [p_diff_=.406] |
| Santiago-Torres  2021  USA[^21^](#_ENREF_21) | QuitGuide  (n=288) | iCanQuit (n=262) | 7-day PPA at 3 months  30-day PPA at 3 months | 17% (n=42/253)  9% (n=22/253) | 26% (n=58/226)  [OR=1.79, p=.012]  15% (n=33/226)  [OR=1.83, p=.041] | 7-day PPA at 6 months.  7-day PPA at 12 months  30-day PPA at 6 months.  30-day PPA at 12 months  Continuous abstinence | 27% (n=70/256)  31% (80/261)  18% (n=46/256)  25% (n=64/261)  10% (n=21/2130 | 35% (n=81/232) [OR=1.41, p=.081]  35% (n=82/231) [OR=1.22, p=.308]  25% (n=57/232) [OR=1.47, p=.089]  29% (n=66/231) [OR=1.19, p=.391]  15% (n=28/185) [OR=1.66, p=.105] |
| Schorling  1997  USA[^22^](#_ENREF_22) | LC (n=304) | BC (n=344) | - | - | - | 30-day PPA CA at 18-months | 6.2% | 9.6% [p_diff_>.05] |
| Sheffer  2004  USA[^23^](#_ENREF_23) | - | n=1644 | “Quit tobacco on discharge” (post-treatment), and at 3-months post discharge, compared to all patients. | - | Post treatment and program, CC: 74.0% (all patients=42%) [p=NR]  3-months post discharge, CC: 42.0% (all patients=30.0%) [p=NR] | - | - | - |
| Sheffer  2009  USA[^24^](#_ENREF_24) | - | n=2350 | 7-day PPA/ sustained abstinence at 3-months post intervention completion | - | CC: 28.6% [p=NR]  ITT: 19.0% [p=NR] | 7-day PPA CA at 12-months | - | CC: 24.3% [p=NR]  ITT: 13.0% [p=NR] |
| Stoops  2009  USA[^25^](#_ENREF_25) | YC (n=33) | AC (n=35) | Biochemically verified (CO<4ppm) at weeks (W) 1,2,3,4,5,6 | CO(-) % samples:  W1 (18.4%)  W2 (8.7%)  W3 (6.7%)  W4 (10.0%)  W5 (11.3%)  W6 (12.6%) | CO(-) % samples:  W1 (30.4%) [p<.05]  W2 (39.0%)  [p<.05]  W3 (39.8%) [p<.05]  W4 (36.9%) [p<.05]  W5 (39.8%) [p=NR]  W6 (33.9%) [p=NR] | - | - | - |
| Wong  2004  Australia[^26^](#_ENREF_26) | - | n=18 | Self-reported PPA at intervention completion (2-months), and 3-months post intervention completion | - | 2-month CC: 38.9% (n=7/18) [p=NR;  3-months post CC: 38.9% (n=7/18) [p=NR] | - | - | - |

**Note.** Where frequencies are not directly reported in original articles, percentages are provided. PPA: Point prevalence abstinence. CA: Continuous abstinence. CC: Complete Case. ITT: Intention-to-treat. NR: Not reported. Significance values for difference refer to pre-post or between conditions comparison and are reported as cut-offs when exact values were not available.

**References**

1. Adams K, Rumbiolo D, Charles S. Evaluation of Rumbalara's' No More Dhonga' short course in giving up smokes. *Aborig Isl Health Work J*. 2006;30(5):20-21.

2. Azor Hui Sk, Nazir N, Faseru B, Ellerbeck EF. Ongoing self‐engagement in quit attempts and cessation outcomes among rural smokers who were unable to quit after 2 years of repeated interventions. *J Rural Health*. 2013;29(1):106-112.

3. Bailey BA. Effectiveness of a pregnancy smoking intervention: the Tennessee intervention for pregnant smokers program. *Health Educ Behav*. 2015;42(6):824-831.

4. Bottorff JL, Seaton CL, Viney N, Stolp S, Krueckl S, Holm N. The Stop Smoking Before Surgery Program: Impact on awareness of smoking-related perioperative complications and smoking behavior in Northern Canadian communities. *J Prim Care Community Health*. 2016;7(1):16-23.

5. Breen RJ, Frandsen M, Ferguson SG. Incentives for smoking cessation in a rural pharmacy setting: The Tobacco Free Communities program. *Aust J Rural Health*. 2021;29(3):455-463. doi:10.1111/ajr.12724

6. Britton GRA, Brinthaupt J, Stehle JM, James GD. The effectiveness of a nurse-managed perinatal smoking cessation program implemented in a rural county. *Nicotine Tob Res*. 2006;8(1):13-28.

7. Bullock L, Everett KD, Mullen PD, Geden E, Longo DR, Madsen R. Baby BEEP: A randomized controlled trial of nurses’ individualized social support for poor rural pregnant smokers. *Matern Child Health J*. 2009;13(3):395-406.

8. Byaruhanga J, Paul CL, Wiggers J, et al. The short-term effectiveness of real-time video counselling on smoking cessation among residents in rural and remote areas: An interim analysis of a randomised trial. *J Subst Abuse Treat*. 2021;131:108448. doi:10.1016/j.jsat.2021.108448

9. Carlson LE, Lounsberry JJ, Maciejewski O, Wright K, Collacutt V, Taenzer P. Telehealth-delivered group smoking cessation for rural and urban participants: Feasibility and cessation rates. *Addict Behav*. 2012;37(1):108-114.

10. Ellerbeck EF, Mahnken JD, Cupertino AP, et al. Effect of varying levels of disease management on smoking cessation: A randomized trial. *Ann Intern Med*. 2009;150(7):437-446.

11. Ferketich AK, Pennell M, Seiber EE, et al. Provider-delivered tobacco dependence treatment to Medicaid smokers. *Nicotine Tob Res*. 2014;16(6):786-793.

12. Gould GS, Watters T. Are single-session smoking cessation groups a feasible option for rural Australia?–Outcomes from a pilot study. *J Smok Cessat*. 2015;10(2):135-140.

13. Hancock L, Sanson-Fisher R, Perkins J, McClintock A, Howley P, Gibberd R. Effect of a community action program on adult quit smoking rates in rural Australian towns: the CART project. *Prev Med*. 2001;32(2):118-127.

14. Harris M, Reynolds B. A pilot study of home‐based smoking cessation programs for rural, Appalachian, pregnant smokers. *J Obstet Gynecol Neonatal Nurs*. 2015;44(2):236-245.

15. Horn K, Dino G, Kalsekar I, Massey CJ, Manzo-Tennant K, McGloin T. Exploring the relationship between mental health and smoking cessation: A study of rural teens. *Prev Sci*. 2004;5(2):113-126.

16. Ivers RG, Farrington M, Burns CB, et al. A study of the use of free nicotine patches by Indigenous people. *Aust N Z J Public Health*. 2003;27(5):486-490.

17. Marley JV, Atkinson D, Kitaura T, et al. The Be Our Ally Beat Smoking (BOABS) study: A randomised controlled trial of an intensive smoking cessation intervention in a remote aboriginal Australian health care setting. *BMC Public Health*. 2014;14(1):1-10.

18. Northridge ME, Vallone D, Xiao H, et al. The importance of location for tobacco cessation: Rural–urban disparities in quit success in underserved West Virginia counties. *J Rural Health*. 2008;24(2):106-115.

19. Reynolds B, Harris M, Slone SA, et al. A feasibility study of home-based contingency management with adolescent smokers of rural Appalachia. *Exp Clin Psychopharmacol*. 2015;23(6):486.

20. Richter KP, Shireman TI, Ellerbeck EF, et al. Comparative and cost effectiveness of telemedicine versus telephone counseling for smoking cessation. *J Med Internet Res*. 2015;17(5):e113.

21. Santiago-Torres M, Mull KE, Sullivan BM, Ferketich AK, Bricker JB. Efficacy of an acceptance and commitment therapy-based smartphone application for helping rural populations quit smoking: Results from the iCanQuit randomized trial. *Prev Med*. 2022;157:107008. doi:10.1016/j.ypmed.2022.107008

22. Schorling JB, Roach J, Siegel M, et al. A trial of church-based smoking cessation interventions for rural African Americans. *Prev Med*. 1997;26(1):92-101.

23. Sheffer CE, O'Bannon R. Initial results from the Arkansas Smoking Cessation Network program. *J Ark Med Soc*. 2004;100(10):356-360.

24. Sheffer CE, Stitzer M, Payne TJ, Applegate BW, Bourne D, Wheeler JG. Treatment for tobacco dependence for rural, lower-income smokers: Outcomes, predictors, and measurement considerations. *Am J Health Promot*. 2009;23(5):328-338.

25. Stoops WW, Dallery J, Fields NM, et al. An internet-based abstinence reinforcement smoking cessation intervention in rural smokers. *Drug Alcohol Depend*. 2009;105(1-2):56-62.

26. Wong BS, Fraser J. The use of Buproprion for smoking cessation in rural NSW. *Aust J Rural Health*. 2004;12(2):38-39. doi:10.1111/j.1038-5282.2004.00554.x
